# NF-κB signaling and the tumor microenvironment in osteosarcoma: implications for immune evasion and therapeutic resistance

**DOI:** 10.3389/fimmu.2025.1518664

**Published:** 2025-01-30

**Authors:** Shaoyan Shi, Xuehai Ou, Chao Liu, Rui Li, Qianjin Zheng, Leiming Hu

**Affiliations:** Department of Hand Surgery, Honghui Hospital, Xi’an Jiaotong University, XI’an, China

**Keywords:** osteosarcoma, NF-κB signaling, tumor microenvironment, immune evasion and, malignant bone tumor

## Abstract

Osteosarcoma, a highly aggressive malignancy with a generally poor prognosis, is characterized by tumor cells’ ability to evade immune responses and resist treatment. The nuclear transcription factor NF-κB signaling pathway is crucial in regulating inflammatory and immune reactions. It occupies a central position in the development of the osteosarcoma tumor microenvironment. This research aimed to explore how NF-κB influences the recruitment and polarization of tumor-associated macrophages and myeloid-derived suppressor cells, both of which contribute to immunosuppression. Furthermore, NF-κB facilitates immune surveillance evasion in osteosarcoma cells by altering the expression of immune checkpoint molecules, such as PD-L1. It also enhances tumor cell resistance to chemotherapy and radiotherapy by activating anti-apoptotic signaling pathways and exacerbating treatment-induced inflammation. Potential therapeutic approaches include using NF-κB inhibitors, possibly in combination with immune checkpoint inhibitors, to overcome tumor cell resistance mechanisms and reshape antitumor immune responses. A thorough examination of NF-κB’s role in osteosarcoma development is expected to yield novel clinical treatment strategies, and significantly improve patient prognosis by targeting this key signaling pathway.

## 1 Introduction

Osteosarcoma (OS) is the most prevalent primary malignant bone tumor, predominantly affecting adolescents and young adults. The incidence of osteosarcoma reaches its peak during periods of rapid bone growth, particularly in individuals between 10 and 25 years of age (1, 2). This neoplasm is characterized by malignant cells that produce immature bone or osteoid tissue (3, 4). Despite its relatively low overall incidence, osteosarcoma remains the leading cause of cancer-related mortality among adolescents. This is primarily attributed to its highly aggressive nature and early propensity for metastasis, particularly to the lungs, which significantly complicates treatment and prognosis. The clinical manifestations of osteosarcoma include localized pain, swelling, and occasionally pathological fractures. Due to to its aggressive nature, approximately 20% of patients present with metastatic disease at the time of diagnosis, with the lungs being the most common site of dissemination (5, 6). The prognosis for osteosarcoma patients largely dependent on the presence or absence of metastasis at diagnosis. For patients with localized disease, the current standard treatment—comprising neoadjuvant chemotherapy, surgical resection, and postoperative chemotherapy—yields a 5-year survival rate of approximately 60-70% (7–9). However, for patients with metastatic disease, the survival rate significantly decrease to approximately 20-30%, despite advancements in surgical techniques and chemotherapy regimens.

One of the most significant challenges in treating osteosarcoma is its inherent resistance to chemotherapy and radiotherapy (10, 11). Despite chemotherapy’s crucial role in managing osteosarcoma, utilizing medications such as doxorubicin, methotrexate, and cisplatin, many patients develop either innate or acquired resistance. Furthermore, the heterogeneous nature of osteosarcoma complicates treatment strategies, as various tumor cell subgroups may display different genetic and molecular characteristics, leading to varied drug responsiveness. Another significant challenge is the high frequency of metastasis, especially to the lungs. Upto 40% of osteosarcoma patients experience pulmonary metastases at some stage of disease progression, which is linked to a poor prognosis (12, 13).

The tumor microenvironment (TME) plays a crucial role in the initiation, progression, and therapeutic resistance of various cancers, including osteosarcoma (14, 15). The TME consists of a complex network of cellular and molecular components that interact with tumor cells, influencing tumor growth, immune system evasion, and treatment response. Principal cellular constituents of the TME include immune cells, such as tumor-associated macrophages (TAMs), myeloid-derived suppressor cells (MDSCs), T-cells, and natural killer (NK) cells. Furthermore, stromal cells—including cancer-associated fibroblasts (CAFs), endothelial cells, and mesenchymal stem cells (MSCs)—play essential roles within this milieu (16, 17). These cells secrete diverse cytokines, chemokines, growth factors, and extracellular matrix (ECM) components, all of which contribute to the tumor-promoting environment (18, 19).

Within osteosarcoma, the TME plays a crucial role in facilitating immune evasion, tumor growth, and metastasis. Osteosarcoma cells secrete factors that recruit immunosuppressive cells, including tumor-associated macrophages (TAMs) and MDSCs, which inhibit the anti-tumor immune response and promote tumor development (3, 20). TAMs in the osteosarcoma microenvironment are frequently polarized towards the M2 phenotype, which is associated with tissue remodeling, angiogenesis, and immunosuppression, all of which contribute to tumor progression. MDSCs, a heterogeneous population of immature myeloid cells, are also recruited to the osteosarcoma TME, where they suppress T-cell activity and enhance immune evasion by the tumor (21, 22). The TME in osteosarcoma also plays a significant role in promoting resistance to therapies. Tumor cells within the microenvironment are often exposed to hypoxic conditions, which can induce resistance to chemotherapy and radiotherapy. Hypoxia-inducible factors (HIFs) within the TME activate signaling pathways that enable tumor cells to adapt to low oxygen levels, enhancing their survival and proliferation (23–25). Furthermore, cancer-associated fibroblasts (CAFs) contribute to resistance by secreting factors that promote tumor cell survival and by remodeling the extracellular matrix (ECM) to create a physical barrier to drug delivery.

The nuclear factor-kappa B (NF-κB) family of transcription factors plays a pivotal role in controlling immune and inflammatory responses. NF-κB is activated by numerous stimuli, including cytokines, growth factors, microbial components, and stress signals (26, 27). Upon activation, NF-κB translocates to the nucleus, where it regulates the expression of genes involved in inflammation, cell growth, survival, and immune system regulation. Under physiological conditions, NF-κB signaling is tightly regulated, however, its dysregulation is a characteristic features of many cancers, including osteosarcoma (28, 29). NF-κB signaling is crucial in orchestrating the immune system response to infections and injuries, regulating the pro-inflammatory cytokine production, including that of tumor necrosis factor-alpha (TNF-α), interleukin-1 (IL-1), and interleukin-6 (IL-6), which initiate and maintain inflammatory processes. Furthermore, NF-κB mediates the expression of genes that promote immune cell survival and proliferation, enabling an effective response against pathogens or damaged cells (30, 31). In cancer, NF-κB is frequently aberrantly activated, causing persistent inflammation that promotes tumor progression and facilitates immune evasion. Constitutive NF-κB activation within tumor cells and the surrounding TME stimulates the release of cytokines and chemokines, which recruit immune-suppressive cells such as tumor-associated macrophages (TAMs) and MDSCs to the tumor site. This recruitment fosters an immunosuppressive microenvironment, enabling tumor cells to evade immune surveillance and destruction (32, 33).

In osteosarcoma, NF-κB signaling plays a critical role in promoting tumor progression, immune evasion, and treatment resistance. Numerous osteosarcoma cells exhibit constitutively active NF-κB, causing the increased expression of genes supporting cell survival, proliferation, and metastasis. For example, NF-κB regulates the production of anti-apoptotic proteins such as Bcl-2 and Bcl-xL, which facilitate osteosarcoma cell’s evasion of chemotherapy-induced apoptosis (34, 35). Furthermore, NF-κB-driven inflammation is a significant factor in the immune evasion mechanisms employed by osteosarcoma cells. These cells secrete pro-inflammatory cytokines that recruit TAMs and MDSCs to the TME, where they suppress the anti-tumor immune response (36). TAMs, in particular, are known for secreting immunosuppressive cytokines such as IL-10 and TGF-β, which inhibit the function of cytotoxic T-cells and natural killer cells. This suppression enables osteosarcoma cells to evade immune detection and escape immune surveillance (36).

NF-κB’s significant influence on osteosarcoma is well-documented. By activating NF-κB, genes associated with epithelial-to-mesenchymal transition (EMT) are upregulated, allowing osteosarcoma cells to become invasive and migratory. This mechanism enables tumor cells to spread from their primary location, penetrate nearby tissues, and migrate to distant organs, particularly the lungs. Moreover, NF-κB signaling enhances angiogenesis by upregulating vascular endothelial growth factor (VEGF) expression, thus facilitating the formation of new blood vessels to nourish and sustain tumor growth (37, 38). NF-κB also plays a crucial role in osteosarcoma’s resistance to therapy. When exposed to chemotherapy or radiotherapy, NF-κB activation triggers survival pathways that shield osteosarcoma cells from treatment-induced cell death (38, 39). This mechanism contributes to the development of chemoresistance and radioresistance, which pose significant challenges in effectively treating osteosarcoma.

The NF-κB signaling cascade plays a crucial role in the development of osteosarcoma, influencing tumor progression, immune evasion, and treatment resistance. By modulating inflammatory responses, immune cell recruitment, and cell survival, NF-κB fosters a pro-tumorigenic microenvironment that enables osteosarcoma cells to evade immune surveillance and resist conventional therapies. Elucidating the molecular mechanisms through which NF-κB operates in osteosarcoma is essential for developing targeted therapies that can disrupt this pathway and enhance therapeutic outcomes for patients with this aggressive malignancy.

## 2 NF-κB in shaping the immunosuppressive tumor microenvironment in osteosarcoma

**Figure 1:**
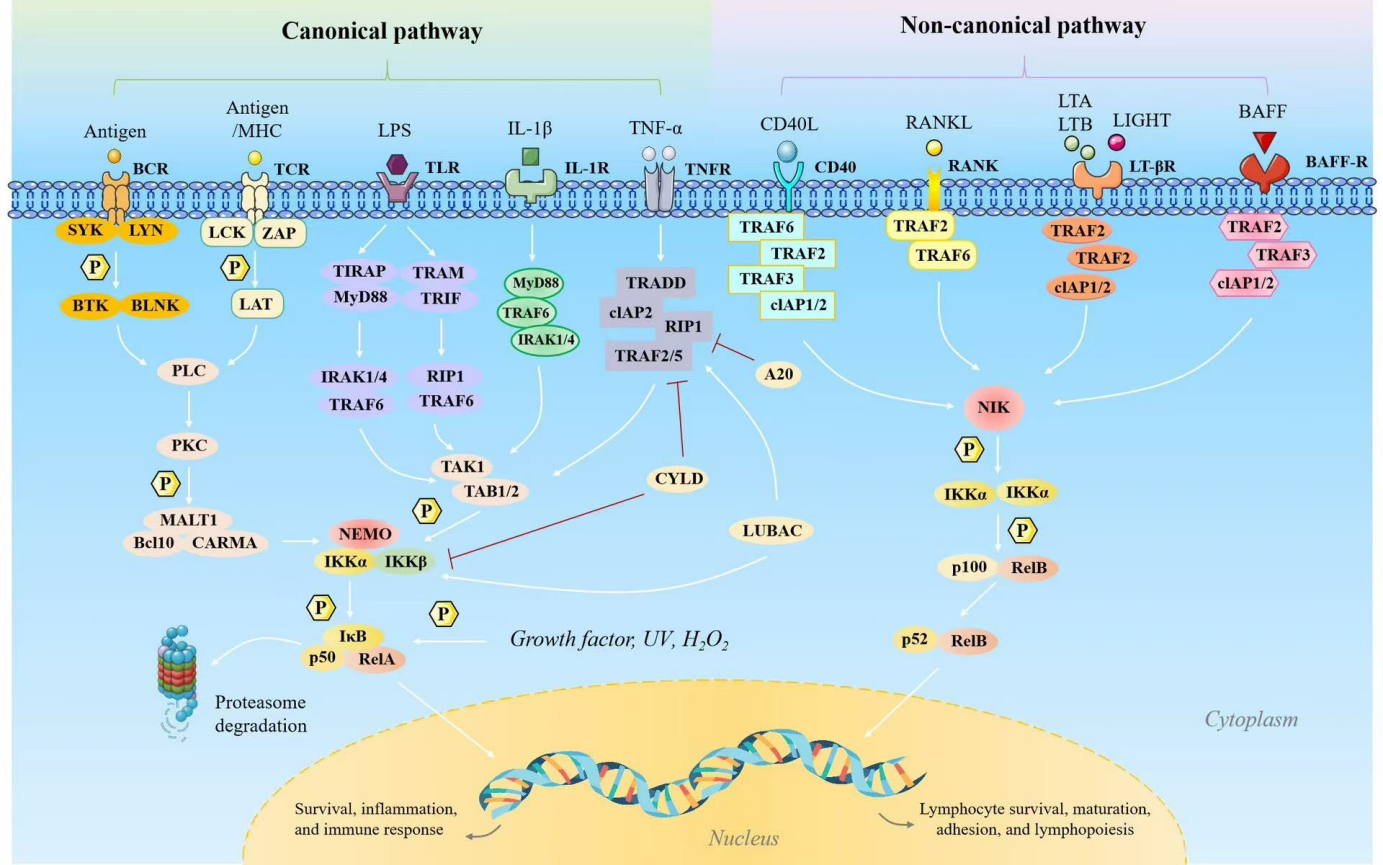
The two NF-κB signaling pathways include the classical (canonical) and alternative (noncanonical) routes. The classical NF-κB signaling pathway is activated by factors such as tumor necrosis factor (TNF), interleukin-1 (IL-1), and Toll-like receptor ligands including lipopolysaccharide (LPS), as well as through the activation of T cell and B cell receptors. This pathway plays a critical role in regulating gene expression associated with inflammation, cell proliferation, survival, epithelial-to-mesenchymal transition, invasion, angiogenesis, and metastasis. However, the alternative NF-κB signaling pathway is initiated by molecules such as lymphotoxin (LT), receptor activator of NF-κB ligand (RANKL), CD40 ligand (CD40L), and B cell-activating factor (BAFF). It primarily induces genes essential for the formation and maintenance of secondary lymphoid structures and associated components within the tumor microenvironment (TME). While LPS can also stimulate the alternative pathway to a lesser extent, LT, RANKL, CD40L, and BAFF retain the classical pathway activation capacity as well.

In osteosarcoma, as in numerous other malignancies, TME constitutes a complex ecosystem comprising only neoplastic cells but also diverse non-cancerous cellular components, including immune cells, fibroblasts, and endothelial cells, all of which interact via cytokines, chemokines, and growth factors. The NF-κB signaling pathway functions as a primary molecular regulator of these interactions, orchestrating various cellular activities in both tumor and immune cells and enhancing the immunosuppressive characteristics of the TME. This immunosuppression is crucial in enabling osteosarcoma cells to evade immune detection and proliferate without unrestrictedly, while also contributing to their resistance to treatment (**Figure 1**). Elucidating the mechanisms by which NF-κB influences the immunosuppressive TME in osteosarcoma is essential for developing more effective therapeutic strategies.

### 2.1 Role of tumor-associated macrophages

Tumor-Associated macrophages (TAMs) constitute one of the most prevalent immune cell populations in the TME of numerous solid tumors, including osteosarcoma. Their presence frequently correlates with poor prognosis due to their role in promoting tumor growth, immune evasion, and metastasis. Chemokines and growth factors, largely regulated by NF-κB signaling, are the primary drivers of TAM recruitment to the osteosarcoma microenvironment (40, 41). Among the various chemokines secreted by osteosarcoma cells, including CCL2 (monocyte chemoattractant protein-1, MCP-1) plays a crucial role. It interacts with CCR2 receptor on circulating monocytes, facilitating their recruitment into the tumor microenvironment (42–44). NF-κB functions as a key regulator of CCL2 expression, and its activation in osteosarcoma cells enhances the production of this chemokine, thus enhancing TAM recruitment. Other NF-κB-regulated factors, such as colony-stimulating factors (CSF1 and CSF2), also contribute to monocyte recruitment and their differentiation into TAMs within the tumor niche (45, 46). This process establishes a feedback loop, wherein the presence of TAMs further augments the immunosuppressive and tumor-promoting characteristics of the osteosarcoma TME.

TAMs demonstrates remarkable adaptability, adopting distinct functional states in response to microenvironmental cues. They are typically categorized into two phenotypes: the pro-inflammatory M1 type and the immunosuppressive M2 type. The NF-κB signaling pathway plays a crucial role in this polarization process. ing signals, such as interferon-gamma (IFN-γ) and lipopolysaccharide (LPS), typically activate the classical NF-κB pathway, promoting the development of M1-like macrophages (47, 48). However, in the osteosarcoma TME, TAMs predominantly shift towards the M2 phenotype, influenced by NF-κB-regulated factors, including IL-10, transforming growth factor-beta (TGF-β), and macrophage colony-stimulating factor (M-CSF) (3, 49). These M2 macrophages secrete anti-inflammatory cytokines and express markers like arginase-1 (ARG1), mannose receptor (CD206), and IL-10, which facilitate tissue remodeling, angiogenesis, and tumor immune evasion. M2-polarized TAMs contribute to an immunosuppressive microenvironment by suppressing the activity of cytotoxic T-cells and NK cells, thus hindering effective immune responses against osteosarcoma cells (50). The transition between M1 and M2 states is not irreversible, and NF-κB signaling plays a complex role in regulating this equilibrium. Persistent NF-κB activation within the TME supports the maintenance of the M2 phenotype by enhancing the production of immunosuppressive cytokines and chemokines, thereby augmenting the tumor-promoting functions of TAMs (51, 52).

TAMs exhibit a multifaceted influence on osteosarcoma progression. When TAMs shift to the M2 phenotype, they facilitate immune evasion by secreting immunosuppressive molecules such as IL-10 and TGF-β. These substances inhibit T-cells and NK cell activation and proliferation, creating a localized immune environment that hinders antitumor responses and enables osteosarcoma cells to evade immune-mediated destruction (53). In addition to their immunosuppressive role, TAMs contribute to tumor growth by producing growth factors like VEGF, which promote angiogenesis, ensuring a continuous supply of oxygen and nutrients to support tumor expansion. The NF-κB signaling pathway is crucial in regulating these tumor-promoting activities, as it governs the expression of genes involved in angiogenesis, ECM remodeling, and cell migration (54, 55). Furthermore, TAMs enhance metastasis by establishing a pre-metastatic niche that supports the survival and proliferation of circulating osteosarcoma cells. Through the secretion of chemokines and growth factors, TAMs prepare distant organs, such as the lungs, to support the colonization of metastatic osteosarcoma cells. This process is tightly regulated by NF-κB, which regulates the expression of key metastasis mediators, including MMPs and chemokines such as CCL2 and CCL5 (56, 57).

Chemokines and growth factors secreted by tumor cells attract these cells to the tumor location, with many of these signals regulated by NF-κB signaling. High concentrations of chemokines such as CCL2, CXCL12, and CXCL5 are produced by osteosarcoma cells, which bind to receptors on MDSCs and stimulate their migration into the TME (58, 59). Once inside the TME, MDSCs encounter NF-κB-driven cytokines like IL-6 and GM-CSF, which aid in their survival and proliferation. The continuous recruitment and maintenance of MDSCs in the osteosarcoma microenvironment is ensured by NF-κB activation in both tumor and stromal cells, where they exert potent immunosuppressive effects (60, 61).

T-cell function is potently inhibited by MDSCs, which effectively suppresses the adaptive immune response against osteosarcoma. MDSCs also deplete essential nutrients, such as L-arginine and cysteine, from the microenvironment, further impairing T-cell function (62). The expression of key enzymes like arginase-1 and inducible nitric oxide synthase (iNOS), is controlled by NF-κB signaling. These enzymes play a vital role in the immunosuppressive actions of MDSCs (62).

NF-κB not only facilitates the recruitment of MDSCs but also plays a crucial role in enhancing their proliferation and survival within the osteosarcoma TME. NF-κB activation in response to pro-inflammatory cytokines such as IL-6 and TNF-α results in the upregulation of survival pathways in MDSCs, enabling their persistence in the TME and continued suppression of antitumor immune responses. Moreover, NF-κB signaling augments the expression of genes responsible for MDSC population expansion, thereby maintaining a persistent immunosuppressive environment that promotes tumor progression. Consequently, NF-κB serves as a critical regulator of MDSC-mediated immune suppression in osteosarcoma.

### 2.2 Other immune components in the TME affected by NF-κB

NF-κB signaling impacts various immune cells in the osteosarcoma TME, including NK cells, regulatory T-cells (Tregs), and dendritic cells (DCs), beyond its effects on TAMs and MDSCs. NK cells, as innate immune cells, essential for tumor cell eradication, can be hindered by NF-κB-regulated cytokines and chemokines in the osteosarcoma TME. For instance, TAM-derived TGF-β and IL-10, both regulated by NF-κB, suppress NK cell cytotoxicity, allowing osteosarcoma cells to evade NK cell-mediated immune surveillance (63, 64). NF-κB signaling also promotes the expansion and recruitment of regulatory T-cells (Tregs), which are responsible for maintaining immune tolerance. In the osteosarcoma TME, Treg further suppress cytotoxic T-cells and NK cells, NF-κB-regulated cytokines like IL-10 and TGF-β playing key roles in enhancing Treg expansion and function, thus contributing to the immunosuppressive environment (65, 66). While NF-κB is vital for dendritic cells (DCs) activation and promoting antitumor immunity, chronic NF-κB activation can result in immunosuppressive DCs that ineffectively prime T-cells and instead promote tolerance. The immunosuppressive network within the osteosarcoma TME is intricate and tightly regulated by NF-κB signaling. The interactions among TAMs, MDSCs, Tregs, and other immune cells establishes a microenvironment that facilitates tumor growth, metastasis, and therapy resistance. At the core of this network, NF-κB directs the production of cytokines, chemokines, and various other factors that uphold the immunosuppressive and tumor-supportive characteristics of the TME.

## 3 NF-κB-Driven inflammation and immune evasion in osteosarcoma

**Figure 2:**
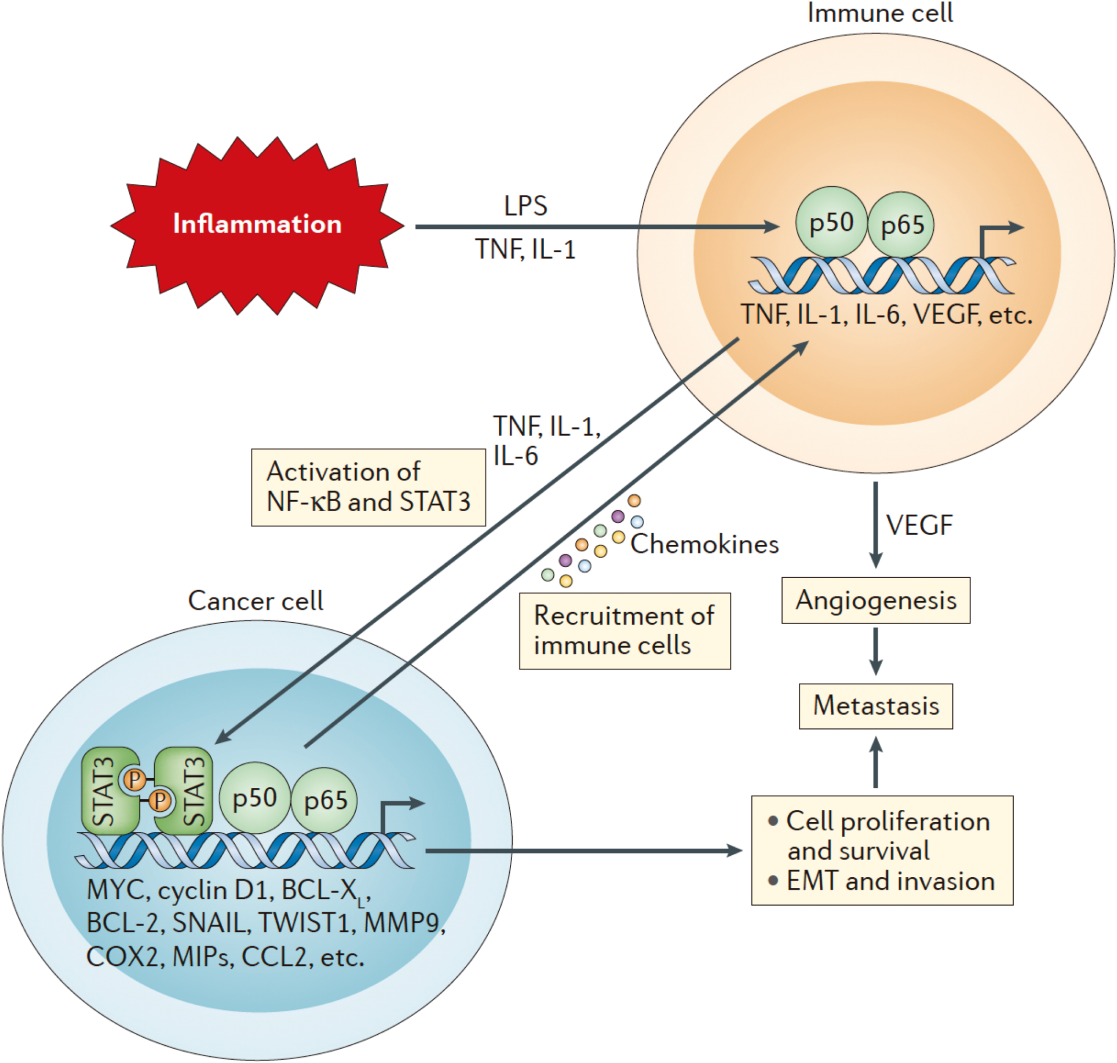
The initial activation of Nuclear factor-κB (NF-κB) in immune cells occurs through tumor necrosis factor (TNF), interleukin-1 (IL-1), and various pathogen-associated or damage-associated molecular patterns. This activation leads to the secretion of pro-inflammatory cytokines, chemokines, and growth factors, including TNF, IL-1, IL-6, and vascular endothelial growth factor (VEGF). These pro-inflammatory signals further stimulate NF-κB and signal transducer and activator of transcription 3 (STAT3) in both cancer cells and components of the TME, enhancing cancer cell proliferation, survival, epithelial-to-mesenchymal transition (EMT), invasion, angiogenesis, and metastasis. Additionally, cancer cells produce chemokines that attract additional immune cells to the tumor microenvironment, thereby sustaining local inflammation and establishing a chronic feedback loop that fosters continuous tumor growth and metastasis.

NF-κB is a pivotal regulator of immune responses and inflammation, contributing significantly to the progression of osteosarcoma by promoting both chronic inflammation and immune evasion (**Figure 2**). The aberrant regulation of NF-κB in osteosarcoma creates a TME that maintains inflammatory conditions, accelerates tumor growth, and enables tumor to evade immune detection. This process encompasses several essential components, including the production of pro-inflammatory cytokines, the upregulation of immune checkpoint molecules, and the onset of cytokine storms that promote tumor progression.

### 3.1 NF-κB activation and production of pro-inflammatory cytokines

A key feature of NF-κB activation in osteosarcoma is the persistent production of pro-inflammatory cytokines, which maintains a chronic inflammatory environment within the TME. This chronic inflammation not only promotes tumor cell proliferation and survival but also inhibits immune responses, allowing uncontrolled tumor expansion. In osteosarcoma, NF-κB regulates two crucial cytokines: interleukin-6 (IL-6) and tumor necrosis factor-alpha (TNF-α). These cytokines play a vital role in establishing and maintaining the chronic inflammatory conditions observed in the osteosarcoma TME. IL-6, a pro-inflammatory cytokine, serves a dual function by supporting both inflammation and tumor progression. NF-κB activation in osteosarcoma results in elevated IL-6 production, which subsequently activates the Janus kinase/signal transducer and activator of transcription (JAK/STAT3) pathway (67, 68). Furthermore, IL-6 promotes the recruitment of immunosuppressive cells (69, 70). TNF-α, another essential pro-inflammatory cytokine, contributes to the maintenance of chronic inflammation in the osteosarcoma TME. NF-κB regulates TNF-α expression, which subsequently activates NF-κB, creating a positive feedback loop that sustains inflammation. TNF-α aids in the recruitment of TAMs and other immunosuppressive cells, facilitating immune evasion (71, 72). It also enhances osteosarcoma cell survival by activating anti-apoptotic pathways, rendering the cells more resistant to treatment.

NF-κB signaling in osteosarcoma creates a chronic inflammatory conditions that fosters a TME conducive to immune evasion through various mechanisms. Persistent inflammation within the TME promotes the recruitment and activation of immunosuppressive cells, such as TAMs, MDSCs, and regulatory T cells (Tregs). These cells inhibit the function of cytotoxic T lymphocytes (CTLs) and NK cells, which are vital for recognizing and eliminating tumor cells. For instance, TAMs secrete IL-10 and transforming growth factor-beta (TGF-β), both of which inhibit CTL activation, allowing osteosarcoma cells to escape immune detection (73, 74). Pro-inflammatory cytokines like IL-6 and TNF-α stimulate the growth of MDSCs, which potently suppress T-cell functionality. MDSCs hinder the activation of CTLs and NK cells, diminishing the immune system’s ability to generate a robust response against the tumor, thereby facilitating osteosarcoma’s evasion of immune surveillance (71, 75). Additionally, the chronic driven by NF-κB can impair the function of antigen-presenting cells (APCs), including dendritic cells (DCs), which are crucial for presenting tumor antigens to T-cells. This dysfunction undermines the immune system’s capacity to recognize and target osteosarcoma cells, further enabling immune evasion (76, 77).

### 3.2 Immune checkpoint molecules and NF-κB

Immune checkpoint molecules are critical regulators of immune responses, maintaining balanced immune activation and ensuring immune tolerance. However, in cancer, tumor cells frequently exploit these molecules to evade immune surveillance. NF-κB plays a crucial role in regulating the expression of immune checkpoint molecules, especially programmed death-ligand 1 (PD-L1), which acts as a primary mediator of immune evasion in osteosarcoma. In osteosarcoma, the activation of NF-κB leads to increased PD-L1 expression on both tumor cells and immune cells within the TME (78, 79). Pro-inflammatory cytokines such as TNF-α and IL-6, regulated by NF-κB, enhance PD-L1 expression, enabling tumor cells to interact with the programmed death-1 (PD-1) receptor on T-cells. This interaction suppresses T-cell activation and induces T-cell exhaustion, thereby inhibiting the immune response against the tumor. By upregulating PD-L1 expression, NF-κB enables osteosarcoma cells in evading immune detection and elimination (80, 81). The suppression of T-cell function through this immune checkpoint mechanisms is one of the primary ways osteosarcoma evades immune clearance.

Immune evasion in osteosarcoma is largely facilitated by the increased expression of immune checkpoint molecules, particularly PD-L1, which is driven by NF-κB-mediated inflammation. NF-κB also regulates the expression of other immune checkpoint molecules, including cytotoxic T-lymphocyte-associated protein 4 (CTLA-4) and T-cell immunoglobulin and mucin domain-3 (TIM-3) (82, 83). These molecules further suppress T-cell activation by inhibiting co-stimulatory signals, thus hindering the immune system’s ability to effectively combat osteosarcoma cells. This immune checkpoint-mediated suppression enables osteosarcoma cells to evade immune surveillance, promoting their continued proliferation and metastasis. The ineffectiveness of T cell-mediated immunity plays a critical role in the progression of osteosarcoma and poses a substantial challenge in the development of effective immunotherapies.

### 3.3 Cytokine storm and feedback loops in tumor promotion

The inflammatory process driven by NF-κB extends beyond initial production of pro-inflammatory cytokines. It also establishes positive feedback loops that perpetuate chronic inflammation and enhance tumor progression. This phenomenon, often referred to as a “cytokine storm,” involves the continuous secretion of cytokines and chemokines, establishing a self-sustaining cycle of inflammation and immune suppression.

A critical feedback mechanism in NF-κB-mediated inflammation involves the interaction between TNF-α and NF-κB itself. When NF-κB is activated, it triggers the production of TNF-α, which further stimulates NF-κB, creating a positive feedback loop that sustains inflammation. This cycle not only aids in osteosarcoma cell survival but also promotes the recruitment of immunosuppressive cells like TAMs and MDSCs, thereby intensifying the immunosuppressive characteristics of the TME. Alongside TNF-α, IL-6 contributes significantly to amplifying NF-κB-driven inflammation by activating the JAK/STAT3 pathway, which can subsequently boost NF-κB signaling (84, 85) This establishes another positive feedback loop that sustains chronic inflammation and supports tumor growth. The continuous activation of these feedback mechanisms ensures that the osteosarcoma TME remains in a state of inflammation, immunosuppression, and favorable conditions for tumor progression.

The cytokine storm triggered by NF-κB-driven inflammation contributes to tumor progression by establishing a TME that enhances tumor cell viability, proliferation, and metastasis. Pro-inflammatory cytokines, such as IL-6 and TNF-α, promote tumor cell survival by activating anti-apoptotic pathways, thereby increasing osteosarcoma cells’ resistance to therapeutic interventions. Moreover, these cytokines stimulate angiogenesis by upregulating vascular endothelial growth factor (VEGF) production, which supports the formation of new blood vessels, which supplies the expanding tumor with essential oxygen and nutrients (86, 87). Additionally, chronic inflammation activates matrix metalloproteinases (MMPs), enzymes that degrade the extracellular matrix, enabling tumor invasion and metastasis. The combined sustained inflammation, immune evasion, and enhanced tumor cell survival makes NF-κB-driven inflammation as a crucial factor in osteosarcoma progression.

## 4 NF-κB’s role in therapeutic resistance in osteosarcoma

One of the most significant challenges in treating osteosarcoma is the development of therapeutic resistance. Despite advancement in surgical techniques, chemotherapy, and radiotherapy, many osteosarcoma patients relapse, often with resistant tumors, resulting in poor long-term survival outcomes, particularly in case of metastatic disease. NF-κB signaling pathway plays a crucial role in the emergence of this resistance by regulating various processes within within TME, including inflammation, cell survival, immune evasion, and resistance to apoptosis. Activation of NF-κB has been associated with both intrinsic and acquired resistance to chemotherapy and radiotherapy in osteosarcoma. This section discusses the mechanisms in which NF-κB contributes to chemoresistance and radioresistance and as well as how inflammation induced by treatment further exacerbates resistance.

### 4.1 NF-κB and chemoresistance

A significant obstacle to successful osteosarcoma treatment is chemoresistance, with NF-κB playing a crucial role by promoting cell survival, reducing apoptosis, and enhancing inflammatory response. Several chemotherapeutic agents commonly used in osteosarcoma treatment, including doxorubicin, cisplatin, and methotrexate, initially induce a good response. However, resistance frequently emerges over time, resulting in treatment ineffectiveness.

The primary mechanism of chemotherapy involves triggering DNA damage, oxidative stress, and subsequent apoptosis in rapidly proliferating tumor cells. Nevertheless, osteosarcoma cells often develop mechanisms to evade chemotherapy-induced cellular death, with NF-κB signaling playing a crucial role in this resistance. NF-κB regulates the expression of various genes associated with cell survival, proliferation, and inflammation, enabling tumor cells to resist the cytotoxic effects of chemotherapy.

In osteosarcoma, chemotherapy triggers NF-κB activation through both the canonical (classical) and non-canonical (alternative) pathways. The canonical pathway involves inflammatory cytokines like TNF-α and interleukin-1 (IL-1) activating IκB kinase (IKK), leading to IκB protein phosphorylation and degradation. These IκB proteins typically restrict NF-κB to the cytoplasm, preventing its nuclear entry (88, 89). Once released, NF-κB translocates into the nucleus and initiates transcription of genes that promote cell survival and inhibit apoptosis. In chemoresistance, NF-κB activation upregulates anti-apoptotic proteins, such as Bcl-2, Bcl-xL, and cellular inhibitors of apoptosis proteins (cIAPs). These proteins counteract key components of both intrinsic and extrinsic apoptotic pathways, protecting osteosarcoma cells from chemotherapy-induced apoptosis. In the intrinsic, mitochondria-mediated pathway, elevated Bcl-2 and Bcl-xL levels prevent cytochrome c release, blocking caspase activation, essential for apoptosis. This mechanism enhances the cellular resistance to chemotherapeutic agents, complicating treatment. In the extrinsic pathway, triggered by death receptor signals like Fas, NF-κB-driven upregulation of cIAPs hinders caspase-8 activation, ultimately preventing apoptosis. Furthermore, NF-κB regulates the expression of genes involved in drug resistance, including multidrug resistance protein 1 (MDR1), which encodes P-glycoprotein, a drug efflux pump that reduces intracellular drug concentrations by actively transporting chemotherapeutic agents out of the cell. MDR1 overexpression in osteosarcoma cells diminishes chemotherapy effectiveness by limiting drug accumulation within tumor cells, thus decreasing its cytotoxic effects.

The NF-κB pathway significantly affected by inflammatory cytokines during chemotherapy, especially in osteosarcoma, where the TME is abundant in pro-inflammatory cytokines such as TNF-α, IL-6, and IL-1. These cytokines trigger NF-κB signaling, which contributes to chemoresistance. TNF-α, in particular, is a potent NF-κB activator and has been demonstrated to protect osteosarcoma cells from apoptosis by enhancing the expression of anti-apoptotic proteins like Bcl-2 and cIAPs. Chemotherapy can also stimulate the release of pro-inflammatory cytokines from both tumor and stromal cells, creating a feedback loop that sustains NF-κB activation and enhances survival signaling. Studies indicate that treatment with chemotherapeutic agents such as doxorubicin or cisplatin can induce the release of TNF-α and IL-6. These cytokines subsequently activate NF-κB and increase anti-apoptotic proteins expression, thereby diminishing chemotherapy’s effectiveness and strengthening the cells’ resistance to treatment. This cycle creates a complex environment for eliminating tumor cells, as the reinforced survival signaling undermines the cytotoxic effects of chemotherapy (90, 91). Moreover, the the NF-κB induced anti-apoptotic proteins not only protect tumor cells from chemotherapy-induced death but also enhance their proliferation and resilience under stressful conditions. This enables osteosarcoma cells to evade the cytotoxic effects of chemotherapy and continue to proliferating, leading to the development of chemoresistance and tumor recurrence.

### 4.2 NF-κB and radioresistance

In the treatment of osteosarcoma, radiotherapy is sometimes employed, especially for inoperable tumors or instances of local recurrence. However, tsimilar to chemotherapy, osteosarcoma cells frequently develop resistance to radiotherapy, which reducing its efficacy. NF-κB plays a crucial role in radioresistance, primarily due to its involvement in regulating DNA repair, cell survival, and inflammatory responses to radiation-induced damage. The mechanism of radiation therapy involves causing DNA damage, particularly double-strand breaks, which activate the DNA damage response (DDR). This response triggers various signaling pathways, including the ataxia-telangiectasia mutated (ATM) kinase pathway, which is essential for NF-κB activation following radiation-induced DNA damage. ATM phosphorylates IκB kinase (IKK), resulting in IκB protein degradation and subsequent NF-κB activation. Upon activation, NF-κB translocate to the nucleus and stimulates the transcription of genes involved in DNA repair, cell survival, and inflammation (92, 93). NF-κB contributes primarily by upregulating DNA repair proteins, such as BRCA1, RAD51, and other components of the homologous recombination (HR) pathway. These proteins facilitate the repair of radiation-induced DNA damage, enabling osteosarcoma cells to survive and recover from the radiation therapy effects. By enhancing DNA repair, NF-κB diminishes the cytotoxic impact of radiation, allowing tumor cells to continue proliferating despite treatment. Furthermore, NF-κB activation following radiation therapy induces the expression of anti-apoptotic proteins like Bcl-2 and Bcl-xL, which protect osteosarcoma cells from radiation-induced apoptosis. This inhibition of apoptosis enables tumor cells to withstand the cytotoxic effects of radiation and survive treatment.

Radiotherapy is known to elicit a pro-inflammatory response in the tumor microenvironment, contributing to radioresistance. Irradiated cells release damage-associated molecular patterns (DAMPs) that activate toll-like receptors (TLRs) on immune cells and TAMs. This activation leads to the release of pro-inflammatory cytokines such as TNF-α, IL-6, and IL-1β, which in turn stimulate NF-κB signaling. resulting pro-survival environment supports the survival and proliferation of irradiated tumor cells. The inflammation induced by radiation also promotes the recruitment of immunosuppressive cells, including TAMs, MDSCs, and regulatory T cells (Tregs). These cells enhance radioresistance by inhibiting anti-tumor immune responses. NF-κB plays a crucial role in this process by regulating the expression of chemokines that attract these immunosuppressive cells to the tumor site, creating an immunosuppressive microenvironment that shields osteosarcoma cells from immune-mediated destruction. Furthermore, NF-κB activation following radiation therapy promotes the production of angiogenic factors like vascular endothelial growth factor (VEGF). This leads to the formation of new blood vessels that supply the tumor with essential oxygen and nutrients, not only aiding tumor survival after radiation but also facilitating the growth and metastasis of osteosarcoma cells.

### 4.3 Therapy-induced inflammation and resistance

Inflammation in the tumor microenvironment, paradoxically triggered by chemotherapy and radiotherapy, can enhance NF-κB activity and promote therapeutic resistance. This therapy-induced inflammation creates a feedback loop that maintains NF-κB activation and promotes tumor survival, further complicating treatment strategies. The cellular stress and DNA damage caused by these therapies result in the release of pro-inflammatory cytokines and DAMPs from tumor and stromal cells. These inflammatory signals activate the NF-κB pathway, stimulating the expression of genes involved in cell survival, inflammation, and immune evasion. In the context of chemotherapy, the release of DAMP activates toll-like receptors (TLRs) and other pattern recognition receptors (PRRs) on immune cells, leading to the production of pro-inflammatory cytokines such as TNF-α, IL-6, and IL-1β. These cytokines activate NF-κB in both tumor and immune cells, establishing a feedback loop that sustains inflammation and promotes tumor survival (94, 95). Moreover, chemotherapy-induced NF-κB activation increases the expression of anti-apoptotic proteins, DNA repair enzymes, and drug efflux pumps, all of which contribute to chemoresistance. Radiotherapy similarly results in the release of pro-inflammatory cytokines and DAMPs, activating NF-κB through the DNA damage response (DDR) and immune signaling pathways. This NF-κB activation aids in DNA repair, inhibits apoptosis, and amplifies the inflammatory response, all factors that contribute to radioresistance. The recruitment of immune-suppressive cells, including TAMs and MDSCs, further exacerbates resistance by creating an immunosuppressive microenvironment that shields tumor cells from immune-mediated destruction.

The activation of NF-κB in response to inflammation induced by therapy promotes the recruitment and activation of immune-suppressive cells within the tumor microenvironment. TAMs, MDSCs, and regulatory T cells (Tregs) are crucial in promoting therapeutic resistance by inhibiting anti-tumor immune responses and aiding tumor survival. When NF-κB is activated in TAMs, it triggers the secretion of pro-inflammatory cytokines, including IL-10 and TGF-β, which suppress the activity of cytotoxic T cells and NK cells. This creates an immunosuppressive microenvironment that shields tumor cells from immune-mediated destruction and supports their survival following therapy (96, 97). Furthermore, NF-κB also facilitates the recruitment of MDSCs, which hinder T cell activation and proliferation, further reducing the efficacy of the anti-tumor immune response. MDSCs secrete immunosuppressive factors such as arginase-1 and reactive oxygen species (ROS), which impair T cell function and enhance tumor survival. Moreover, NF-κB signaling facilitates the recruitment and expansion of Tregs, which inhibit effector T cell activity and promote immune tolerance towards tumor cells. By suppressing the anti-tumor immune response, Tregs contribute to immune evasion and resistance to therapy in osteosarcoma cells (98–100).

## 5 Targeting NF-κB in osteosarcoma: therapeutic opportunities

NF-κB’s pivotal function in promoting inflammation, immune evasion, tumor progression, and therapeutic resistance in osteosarcoma renders it a compelling focus for therapeutic strategies. Considering NF-κB influence on multiple critical processes that enhance osteosarcoma aggressiveness, interventions targeting this pathway could yield substantial therapeutic benefits. Nevertheless, due to NF-κB essential role in normal immune and inflammatory responses, therapy approaches must be meticulously developed to specifically inhibit its tumor-promoting functions without causing excessive immunosuppression or unintended consequences. The subsequent sections explore the potential therapeutic opportunities for targeting NF-κB in osteosarcoma, including combination therapies, boosting the efficacy of immune checkpoint inhibitors, and current research efforts.

### 5.1 Combination therapies involving NF-κB inhibition

Combining NF-κB inhibitors with conventional therapies like chemotherapy is considered one of the most effective approaches to address therapeutic resistance in osteosarcoma. This strategy, which targets NF-κB signaling, has the potential to enhance the efficacy of chemotherapy, overcome resistance, and reduce tumor progression.

Numerous therapeutic agents have been developed to target the NF-κB pathway either directly or indirectly. These include: IκB kinase (IKK) plays a crucial role in regulating the NF-κB pathway by phosphorylating IκB, which leads to its degradation and subsequent NF-κB activation (101, 102). IKK inhibitors prevent this phosphorylation, thus hindering NF-κB activation. BAY 11-7082 and BMS-345541 are examples of IKK inhibitors that have shown potential in preclinical models by suppressing NF-κB activity and reducing tumor growth. However, the main challenge is to specifically target the IKK complex in tumor cells without impacting normal tissues, as NF-κB is essential for normal immune responses. The proteasome is involved in the degradation of IκB, a necessary step for NF-κB activation. By inhibiting the proteasome, drugs like bortezomib can prevent IκB degradation, thereby preventing NF-κB activation. Bortezomib has received FDA-approval for treating multiple myeloma, and its potential use in osteosarcoma is currently under investigation. Research has demonstrated that bortezomib can make osteosarcoma cells more susceptible to chemotherapy by inhibiting NF-κB-mediated survival pathways. Various natural compounds, including curcumin and resveratrol, have been found to inhibit NF-κB signaling in cancer cells (103, 104). Curcumin, a polyphenol extracted from turmeric, suppresses NF-κB by preventing IκB phosphorylation and subsequent NF-κB nuclear translocation. It has shown anti-inflammatory and anti-tumor properties in osteosarcoma preclinical models, reducing tumor growth and enhancing chemotherapy efficacy. Although natural compounds are less specific than targeted drugs, they offer the benefit of reduced toxicity and could be used as adjuncts to other therapies.

Combining NF-κB inhibitors with chemotherapy offers the potential to enhance the cytotoxic effects of chemotherapeutic agents by sensitizing tumor cells to apoptosis and overcoming chemoresistance. Osteosarcoma cells frequently become resistance to chemotherapy by upregulating NF-κB-mediated survival pathways, including the expression of anti-apoptotic proteins like Bcl-2 and Bcl-xL. Suppressing NF-κB can downregulate these survival pathways, rendering tumor cells more susceptible to chemotherapy-induced apoptosis. Preclinical studies have demonstrated that combining IKK inhibitors or proteasome inhibitors with chemotherapy can result in synergistic effects. For instance, using bortezomib in conjunction with doxorubicin has been shown to significantly reduce tumor growth in osteosarcoma models by promoting apoptosis and suppressing NF-κB activity. Likewise, curcumin has been found to enhance the effectiveness of cisplatin in osteosarcoma by inhibiting NF-κB activation and reducing inflammation. In clinical applications, the primary challenge is determining the optimal timing and dosage of NF-κB inhibitors to maximize their effectiveness while minimizing toxicity. The development of targeted delivery systems, such as nanoparticle-based therapies, could help achieve this goal by delivering NF-κB inhibitors specifically to tumor cells, thus reducing off-target effects.

### 5.2 Enhancing immune checkpoint inhibitors via NF-κB modulation

Cancer treatment has been revolutionized by immune checkpoint inhibitors, such as anti-PD-1 and anti-CTLA-4 therapies, which enable the immune system to target tumor cells. However, these treatments have shown limited efficacy in osteosarcoma, partly due to the immunosuppressive microenvironment driven by NF-κB-mediated inflammation. Modulating NF-κB activity may improve the effectiveness of immune checkpoint inhibitors in osteosarcoma. NF-κB is crucial in regulating the expression of immune checkpoint molecules, including PD-L1, on both tumor cells and immune cells within the TME. The interaction between PD-L1 and the programmed death-1 (PD-1) receptor on T-cells leads to T-cell exhaustion and facilitates immune evasion (105, 106). By suppressing NF-κB, it may be possible to reduce PD-L1 expression, thereby enhancing the anti-tumor immune response and improving the efficacy of immune checkpoint inhibitors.

Research in preclinical models has demonstrated the potential of combining NF-κB inhibitors with immune checkpoint blockade. Specifically, using IKK inhibitors alongside anti-PD-1 therapy has been shown to enhance T-cell activation and reduce tumor growth across various cancer models. This approach could be especially effective in osteosarcoma, where NF-κB-induced inflammation facilitates immune evasion and hinders the anti-tumor immune response. Furthermore, inhibiting NF-κB may enhance the infiltration of cytotoxic T-cells into the TME by reducing the recruitment of immunosuppressive cells like TAMs and MDSCs. This could potentially enhance the effectiveness of immune checkpoint inhibitors by creating a more conducive immune environment for T-cell-mediated tumor elimination.

Although inhibiting NF-κB shows potential for enhancing the effectiveness of immune checkpoint inhibitors, it is essential to recognize NF-κB complex rule in modulating both pro-inflammatory and anti-inflammatory responses. In certain cases, complete suppression of NF-κB could hinder immune response by reducing the production of pro-inflammatory cytokines essential for effective T-cell activation. Consequently, a more refined approach may be required, such as specifically targeting particular NF-κB subunits or signaling pathways involved in tumor promotion while preserving the broader immune response. Current studies have concentrated on elucidating the distinct roles of the canonical and non-canonical NF-κB pathways in tumor immunity. Targeting the non-canonical pathway, which is more closely linked to immune evasion and tumor progression, might offer a more targeted strategy for enhancing immunotherapy while preserving the beneficial immune functions of the canonical NF-κB pathway.

### 5.3 Preclinical and clinical trials targeting NF-κB in osteosarcoma

Considering NF-κB therapeutic potential, numerous ongoing preclinical and clinical trials are evaluating the effectiveness of NF-κB inhibitors in treating osteosarcoma. Preclinical studies has shown that these inhibitors can effectively reduce osteosarcoma growth and enhancing the efficacy of other therapies. In particular, experiments using IKK inhibitors and proteasome inhibitors in osteosarcoma models have demonstrated notable reductions in tumor size, decreased metastasis, and enhanced sensitivity to chemotherapy. Additionally, natural compounds such as curcumin have yielded promising results in preclinical models by suppressing NF-κB and inhibiting tumor growth without causing significant toxicity. Some studies have also explored the combining NF-κB inhibitors with immune checkpoint inhibitors, revealing that NF-κB inhibition can enhance the anti-tumor immune response by reducing PD-L1 expression and promoting T-cell infiltration into TME.

Numerous ongoing clinical trials are assessing the safety and effectiveness of NF-κB inhibitors for various cancer, including osteosarcoma. Although most of these studies are in their initial phases, they show potential for the development of novel therapeutic strategies. One such trial is investigating bortezomib, a proteasome inhibitor that suppresses NF-κB activation, in conjunction with chemotherapy for osteosarcoma patients. Preliminary findings indicate that bortezomib may improve chemotherapy effectiveness and reduce tumor size in certain patients (107, 108). Clinical trials are examining the use of IKK inhibitors combined with other therapies. These early stage studies aim to identify the optimal dosing and combination strategies to maximize the therapeutic advantages of NF-κB inhibition. Ongoing clinical studies is also evaluating the use of natural compounds like curcumin and resveratrol in cancer therapy. These investigations seek to determine the safety and efficacy of these compounds in combination with conventional therapies.

## 6 Conclusion

The NF-κB signaling pathway plays a vital role in promoting progression, immune evasion, and therapeutic resistance in osteosarcoma. Acting as a central regulator of inflammatory responses, NF-κB manages the expression of various genes associated with cell survival, proliferation, and immune modulation. In the context of osteosarcoma, abnormal activation of NF-κB contributes to the development of a pro-tumorigenic TME that encourages tumor growth, metastasis, and resistance to conventional therapies such as chemotherapy and radiotherapy.

NF-κB’s impact on osteosarcoma progression is largely due to its role in facilitating immune evasion. The osteosarcoma TME is densely populated by immunosuppressive cells, such as TAMs, MDSCs, and regulatory T cells (Tregs). These cells are recruited and activated by NF-κB-driven pro-inflammatory cytokines and chemokines. This influx creates a highly immunosuppressive environment that reduces the effectiveness of cytotoxic T lymphocytes (CTLs) and NK cells, which are crucial for tumor cell recognition and elimination. By promoting the accumulation of these immunosuppressive cells, NF-κB assists osteosarcoma evade immune detection and clearance, enabling uncontrolled tumor cellsgrowth. Beyond immune evasion, NF-κB plays a crucial role in therapeutic resistance in osteosarcoma. Chemoresistance and radioresistance present significant challenges effectively treating osteosarcoma, with NF-κB plays a central role in both mechanisms. When exposed to chemotherapy-induced stress, NF-κB is activated and stimulates the transcription of anti-apoptotic genes like Bcl-2 and Bcl-xL, which protect osteosarcoma cells from apoptosis. Additionally, NF-κB enhances the expression of drug efflux pumps, such as multidrug resistance protein 1 (MDR1), reducing their effectiveness. In a similar manner, radiation therapy triggers NF-κB activation through the DNA damage response pathway, resulting in increased DNA repair, cell survival, and resistance to radiation-induced cytotoxicity.

The chronic activation of NF-κB establishes a feedback loop of inflammation, wherein pro-inflammatory cytokines like TNF-α and IL-6 continually activate NF-κB, leading to increased production of cytokines and chemokines. This chronic inflammation not only sustains the tumor-promoting activities of the TME but also facilitates the recruitment of additional immunosuppressive cells, further enhancing immune evasion and resistance. Consequently, NF-κB functions acts as a crucial regulator of osteosarcoma progression by influencing various processes, including immune suppression, therapeutic resistance, inflammation, and metastasis. Targeting NF-κB signaling in osteosarcoma offers a potential approach to tackle these obstacles and enhance treatment outcomes.

Although our knowledge of NF-κB’s function in osteosarcoma has grown considerably, there are still many aspects to be investigated, especially regarding the most effective ways to target this pathway for treatment. Several research directions offer potential for advancing osteosarcoma treatment through the modulation of NF-κB signaling.

A major obstacle in targeting NF-κB is its ubiquitous role in normal cellular processes, especially in immune responses and inflammatory processes. Broad inhibition of NF-κB could result in severe adverse effects, including immunosuppression and impaired wound healing. Consequently, future research should concentrate on precisely targeting the specific NF-κB pathways or subunits that are most relevant to osteosarcoma progression. As an illustration, while the classical (canonical) NF-κB pathway is extensively involved in immune responses, the alternative (non-canonical) NF-κB pathway may have a more specific role in regulating tumor-promoting processes like lymphogenesis and metastasis. Focusing on the alternative pathway or specific components of the classical pathway involved in anti-apoptotic signaling might offer a more refined therapeutic approach with fewer side effects.

Despite showing promise in preclinical studies, NF-κB inhibitors like IKK inhibitors and proteasome inhibitors, have not been widely adopted in clinical application for osteosarcoma treatment. To advance their use, more research is required to develop more potent and specific NF-κB inhibitors that can be used alongside conventional therapies such as chemotherapy and radiotherapy. Innovative drug delivery methods utilizing nanotechnology, including nanoparticles and liposomes, could offer a novel approach to delivering NF-κB inhibitors directly to tumor cells while reducing systemic toxicity. Moreover, investigating small-molecule inhibitors that target specific NF-κB pathway components, like IKKβ, or exploring natural compounds with NF-κB inhibitory properties, such as curcumin and resveratrol, could open up new possibilities for therapeutic intervention.

Combining NF-κB inhibitors with existing treatments like chemotherapy, radiotherapy, and immunotherapy represents one of the most encouraging approaches to address therapeutic resistance in osteosarcoma. Synergistic effects have been found in preclinical studies when NF-κB are used alongside chemotherapeutic agents, leading to enhanced apoptosis and reduced tumor growth. To assess the safety and effectiveness of these combined therapies in individuals with osteosarcoma, clinical trials are essential. Furthermore, there is significant potential in combining NF-κB inhibitors with immune checkpoint inhibitors, such as anti-PD-1 and anti-CTLA-4 therapies, for enhancing the anti-tumor immune response. These combination therapies could potentially enhance the effectiveness of immunotherapy in osteosarcoma by diminishing the immunosuppressive impact of NF-κB-mediated inflammation. This is particularly important given the limited success of immunotherapy in clinical trials for osteosarcoma thus far.

Given the diverse nature of osteosarcoma, it is likely that not all tumors are equally dependent on NF-κB signaling for their survival and progression. Consequently, future research should aim to discover biomarkers that can predict which patients are most likely to respond positively to NF-κB-targeted therapies. This personalized medicine approach would enable patient classification based on their tumor’s molecular characteristics, leading to more tailored and effective treatment strategies. For instance, tumors exhibiting high levels of NF-κB activation or expression of NF-κB-regulated genes, such as Bcl-2 or PD-L1, might show a better response to NF-κB inhibitors. The identification of these biomarkers could pave the way for more precise and effective therapeutic interventions.

To maximize the effectiveness of NF-κB-targeted therapies in osteosarcoma, it is essential to further investigate the complex interactions between NF-κB signaling and TME. The osteosarcoma TME is a dynamic ecosystem consisting of tumor cells, immune cells, stromal cells, and extracellular matrix components, all of which are affected by NF-κB signaling. Elucidating how NF-κB regulates the actions of various cell types within the TME is crucial for developing more potent therapies. As previously noted, NF-κB plays a pivotal role in shaping the immunosuppressive environment in osteosarcoma. Through its regulation of TAMs, MDSCs, and Tregs, NF-κB facilitates immune evasion and reduce the effectiveness of immunotherapies. Future research should focus on uncovering the precise molecular mechanisms through which NF-κB governs immune cell function within the TME. For instance, determining the signaling cascades that link NF-κB activation to TAM recruitment or the upregulation of immune checkpoint molecules such as PD-L1 could provide valuable insights for developing combination therapies that target both NF-κB and immune checkpoints. In addition to immune cells, stromal cells like cancer associated fibroblasts (CAFs) and endothelial cells also contribute to the NF-κB-mediated tumor-promoting environment in osteosarcoma. CAFs generate extracellular matrix components and secrete pro-inflammatory cytokines, thus supporting tumor growth and metastasis. Endothelial cells, activated by NF-κB, enhance angiogenesis, supplying the tumor with the necessary blood supply for continued growth. Gaining insight into how NF-κB mediates the interactions between tumor cells and stromal cells could lead to the development of therapies that target the entire tumor ecosystem, rather than solely focusing on the tumor cells. An additional critical area of investigation is exploring of how osteosarcoma cells acquire resistance to NF-κB inhibitors. Similar to how tumors develop resistance to chemotherapy through the upregulation of drug efflux pumps or mutations in target proteins, osteosarcoma cells may become resistant to NF-κB inhibitors by activating alternative signaling pathways. Identifying these resistance mechanisms will be crucial for designing second-line therapies to overcome resistance and provide more lasting responses.

NF-κB is a central mediator of osteosarcoma progression, immune evasion, and therapeutic resistance, rendering it a crucial focus for future therapeutic approaches. Through its influence on inflammation, inhibition of apoptosis, and promotion of immune suppression, NF-κB facilitates the aggressive nature of osteosarcoma and reduces the efficacy of standard treatments. Focusing on NF-κB presents promising therapeutic opportunities, such as developing specific NF-κB inhibitors, integrating therapies with chemotherapy and immunotherapy, and developing personalized treatment approaches based on tumor-specific biomarkers. As the investigation into NF-κB’s role in osteosarcoma progresses, we must enhance our comprehension of how this pathway interacts with the TME and contributes to resistance mechanisms. By tackling these issues, it may be feasible to develop more potent and durable therapies that enhance the prognosis for patients with this aggressive and often fatal disease.
